# Effect on White Grape Must of Multiflora Bee Pollen Addition during the Alcoholic Fermentation Process

**DOI:** 10.3390/molecules23061321

**Published:** 2018-05-31

**Authors:** Antonio Amores-Arrocha, Ana Roldán, Ana Jiménez-Cantizano, Ildefonso Caro, Víctor Palacios

**Affiliations:** Department of Chemical Engineering and Food Technology, Faculty of Sciences, University of Cadiz, Agrifood Campus of International Excellence (ceiA3), IVAGRO, P.O. Box 40, 11510 Puerto Real, Cadiz, Spain; ana.roldan@uca.es (A.R.); ana.jimenezcantizano@uca.es (A.J.-C.); ildefonso.caro@uca.es (I.C.); victor.palacios@uca.es (V.P.)

**Keywords:** *Saccharomyces cerevisiae*, bee pollen, yeast-assimilable nitrogen, alcoholic fermentation, white wine

## Abstract

The aim of the present study was to compare and analyze the impact of using bee pollen doses (0.1, 0.25, 1, 5, 10 and 20 g/L) as activator in the alcoholic fermentation process of Palomino fino and Riesling wines. In this regard, its influence on the musts composition, the fermentative kinetics, the evolution of the populations of *Saccharomyces cerevisiae*, the evolution of yeast-assimilable nitrogen and physico-chemical characteristics of final wines has been analyzed. Bee pollen addition produces significant increases in yeast-assimilable nitrogen and maximum yeasts population and exponential velocity reached during alcoholic fermentation. Bee pollen showed an important effect on yeast survival during the death phase. Final wines showed significantly increase in volatile acidity above doses higher than 10 g/L and Comisión Internacional de L’Eclairage parameters (CIELab), color intensity and Abs 420 nm, from 1 g/L. Therefore, pollen could be used as fermentative activator for the alcoholic fermentation of white wines applying doses below of 1 g/L.

## 1. Introduction

During alcoholic fermentation, wine yeasts have different nutritional requirements in different fermentation stages [1,2]. The lack of nutrients and the presence of undesirable substances, such as pesticide residues or antibiotics, can affect the development of yeasts and lead to the slowdown or even total stop of fermentation [1,3,4]. The origin of this lack of nutrients is almost always associated with maturation problems due to a lack of adaptation to the terroir of the varieties or to adverse climatic conditions [5,6]. Due to climatic change, the sugar content in the grape can increase while the acidity decreases, causing musts with potentially high alcohol content and high pH, which are able to affect the quality of the wines [7]. Nutritional deficiencies in musts, such as a lack of yeast-assimilable nitrogen (YAN) [8], and/or vitamins and micronutrients [9,10], can affect the yeast defense system during fermentation and, therefore, their cellular viability [11]. In addition, these changes affect not only the fermentation process but also the sensorial profile of the final wines [12,13].

YAN includes ammoniacal nitrogen, amino acids, small peptides and nitrogen that can be easily assimilated by yeast. The assimilable nitrogen in grape juice contains ammonium and amino acids in similar proportions [14]. YAN is essential for the optimal growth and development of yeast, and levels above 140 mg/L are the minimum amount required for the complete alcoholic fermentation of the grape must [15,16,17]. Sometimes these nutritional requirements are more important for yeasts population development [16,18], especially when used in commercial vinification strains of active dry wine yeast (ADWY), which requires exact levels of YAN for its correct adaptation to the fermentation conditions during the latency phase [18,19]. Certain varieties of grapes are not very rich in YAN and occasionally present maturation problems [5,6], which makes it necessary the use fermentation activators. The increase in the YAN levels by the addition of fermentative activators produces an increase in fermentation speed and an increase in biomass, in addition to intervening in the yeast metabolism and generating volatile and non-volatile metabolites, which can affect the sensory profile of the wines [16,20,21].

In the current oenological market, there is a wide variety of products designed to supply these nutritional deficiencies. Most of the time, musts are dosed with extracts based on amino acids and ammonium, which, in many cases, combine to improve fermentative kinetics [21,22,23], while vitamins improve cell multiplication and adaptation to fermentative conditions (thiamine, biotin, pantothenic acid), and the use of compounds, such as ergosterol and oleanolic acid, aid under anaerobic conditions [24].

Bee pollen is a natural product that is obtained from hives, and it is rich in carbohydrates, proteins, lipids, minerals, vitamins [25,26], essential amino acids (proline, aspartic acid, phenylalanine and glutamic acid) [27], essential fatty acids (linoleic and linolenic acid), sterols, phospholipids and carotenoids [28] and polyphenols [29]. Previous work on meads showed that the addition of very high doses of bee pollen in honey produced a significant YAN contribution [30]. Bee pollen addition reduced the lag phase compared to other activators and increased the cell multiplication of mead, avoiding the metabolic deviations of yeasts during alcoholic fermentation [31]. High doses of bee pollen at 10 and 20 g/L, significantly altered the sensory profile of Palomino fino and Riesling wines, compared to low doses [32].

The aim of the present study is to compare and analyze the impact of using bee pollen as activator in alcoholic fermentation process of Palomino fino and Riesling musts. In this regard, its influence on the composition of the must, the fermentative kinetics, the evolution of the populations of *Saccharomyces cerevisiae*, the evolution of yeast-assimilable nitrogen and physico-chemical characteristics of final wines has been analyzed.

## 2. Results

### 2.1. Influence of Bee Pollen Addition on Grape Must Composition

Table 1 and Table 2 show the physico-chemical parameters of Palomino fino and Riesling grape musts after the addition of different multiflora bee pollen doses and the control, respectively. The addition of pollen does not significantly affect must Beaumè degree (°Bè) density and therefore its sugar concentration, except for the dose of 10 and 20 g/L, where there is a slight increase of 3 and 10 g/L respectively in Palomino fino must and 6 and 9 g/L for Riesling must, respect to their control. These results are very similar to the studies of honey must [30], which estimated a sugar contribution of 0.4 g per each gram of pollen added.

Regarding pH, values are similar to the control for all the pollen doses (Table 1 and Table 2), which was expected, given the high buffering capacity of grape must [33]. Likewise, bee pollen has not effect on free Sulphur concentration. Free SO_2_ levels are similar to the control in all cases, which indicates that studied range doses of bee pollen does not yield substances susceptible to combination with free SO_2_.

On the other hand, yeast-assimilable nitrogen effect of pollen is remarkable. YAN progressively increases with the pollen dose for both varieties with linear correlation values of r^2^ = 0.94 and r^2^ = 0.80, for Palomino fino and Riesling, respectively. YAN percentage increase with pollen dose is slightly higher in the Palomino fino (between 3% and 44%), which presents a YAN of 140 mg/L, compared to the Riesling (between 2% and 30%), which presents a lower YAN of 128 g/L. This suggests that extraction and solubilization of nitrogenous substances from pollen could be influenced by the initial grape must pH regardless of its initial YAN concentration.

### 2.2. Influence of Bee Pollen Values on Alcoholic Fermentation Kinetics

**Figure 1:**
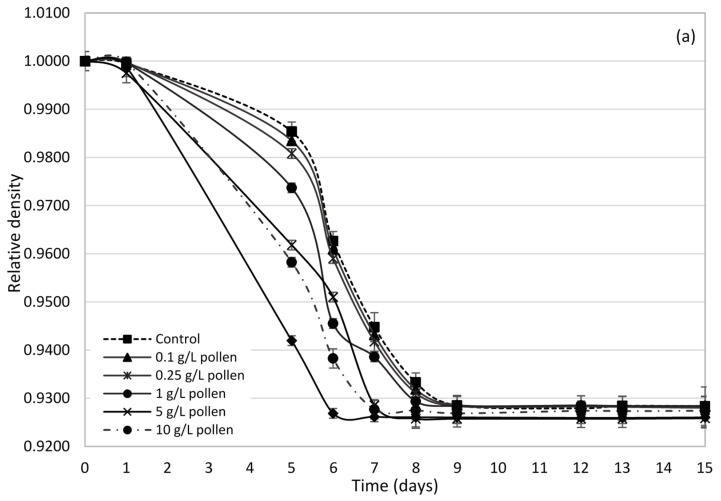

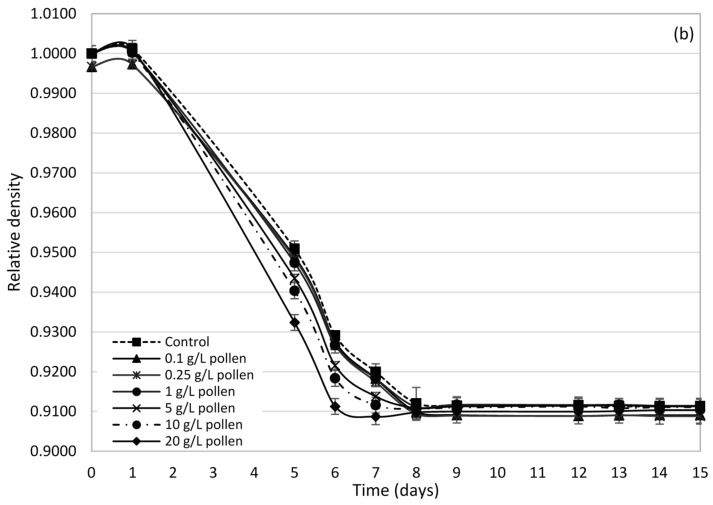
Relative density evolution in Palomino fino (**a**) and Riesling (**b**) grape musts during alcoholic fermentation with the addition of different bee pollen doses.

**Figure 2:**
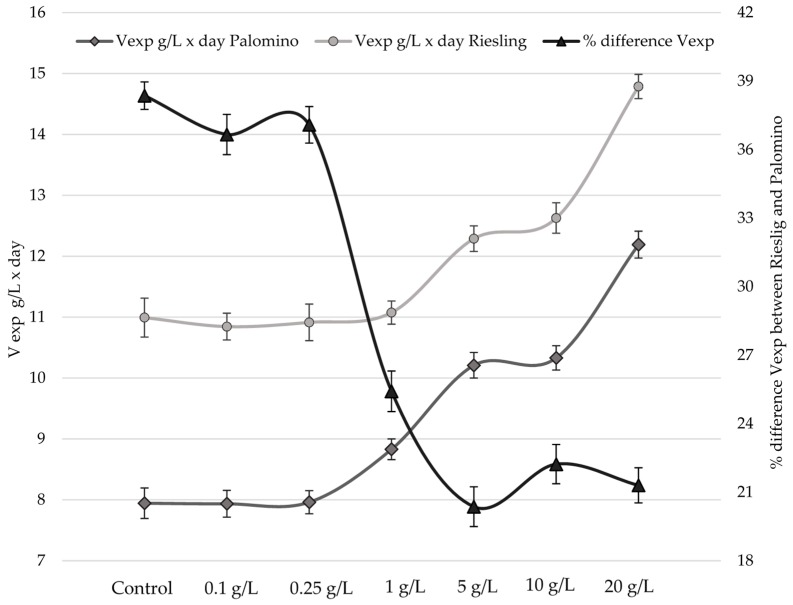
Fermentation rate in the exponential phase (V_exp_).

Figure 1a,b represent the relative density evolution during the alcoholic fermentation processes of Palomino fino and Riesling grape musts, respectively, with different doses of pollen and the control. Generally, except for 20 g/L dose, density slopes fall and are more pronounced and similar to each other in Riesling (Figure 1b) than in the Palomino (Figure 1a). Figure 2 shows that speed during the exponential phase of growth (V_exp_) in Riesling musts is 28% higher than Palomino musts, although from 1 g/L dose these differences begin to be less pronounced. This behaviour cannot be attributed to YAN, since Palomino musts are superior for all YAN cases compared to Riesling must (Table 1 and Table 2). In fact, Riesling musts have no fermentation problems, even those with YAN values below 140 g/L (control and low doses). Some authors indicate that a YAN of 140 mg/L is the minimum necessary to complete and not have yeast growth problems during alcoholic fermentation (AF) [15,17]. Speed difference observed between varieties is due to the initial sugar concentration of musts, directly related to the grape maturation degree at the harvesting time [17]. While Riesling musts have a sugar concentration of 189 g/L, Palomino musts have a concentration of 165 g/L.

Figure 2 shows that V_exp_ of Palomino musts with low pollen doses (from 0.1 to 0.25 g/L) and Riesling low and intermediate (1 g/L) doses cases, are similar to their controls. However, from 1 g/L (Palomino) and 5 g/L (Riesling), V_exp_ increases significantly (ANOVA *p* < 0.05) and proportionally with the dose of bee pollen, as well as when both varieties musts reach YAN levels above 150 mg/L (Table 1 and Table 2).

An important contribution of YAN in alcoholic fermentation with bee pollen can compensate in part for the lack of sugar substrate from the kinetic point of view. Palomino must V_exp_, at 20 g/L with a YAN of 201 mg/L, exceeds the control values and the low and intermediate doses corresponding to Riesling must (Figure 2). Several authors have corroborated that YAN increase in musts produces an increase in the fermentative speed [33,34]. Similarly, high pollen doses addition in honey musts [30] produced a YAN increase, and thus significant and similar increase in the fermentation speed.

### 2.3. Yeast Population Development during AF

**Figure 3:**
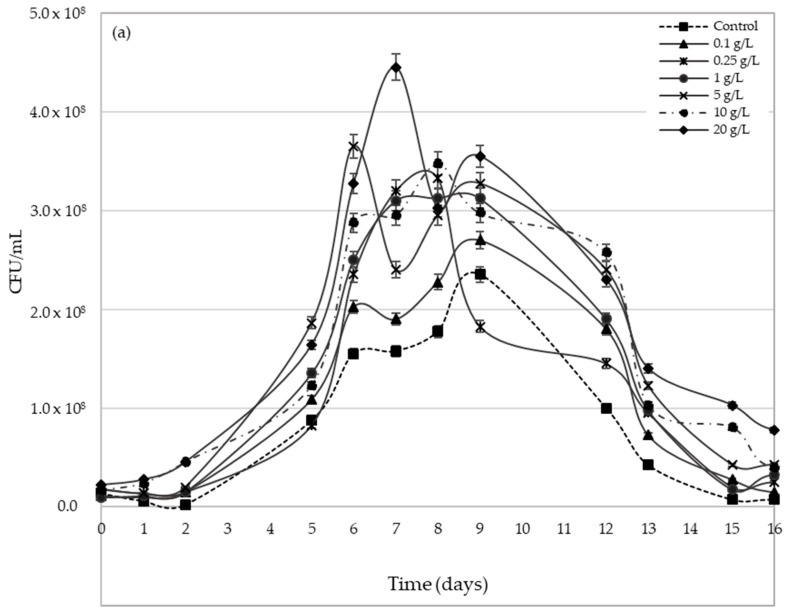

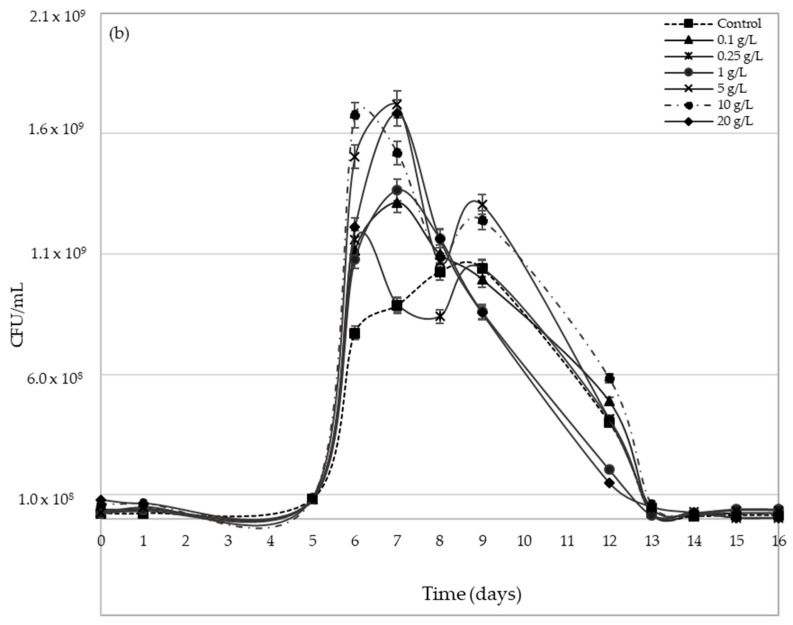
Evolution of the viable biomass of *Saccharomyces cerevisiae* yeasts during the alcoholic fermentation process of Palomino fino (**a**) and Riesling (**b**) grape must with bee pollen doses. The results are the mean ± SD of three repetitions.

Figure 3 shows *Saccharomyces cerevisiae* viable yeasts evolution during the AF process. Both varieties show very different behaviours. In Palomino fino, lag phase lasts approximately 2 days, whereas Riesling showed of 5 days’ lag phase (Figure 3a,b) for all pollen doses and the control. Lag phase elongation in Riesling can be due mainly to its higher sugar concentration, which leads to a longer adaptation time for the yeast inoculum [35].

Regarding fermentative kinetics (Figure 1), it is again observed the variation range or interval of yeasts populations with pollen dose is more remarkable in Palomino musts, possibly because it has higher YAN values (Table 1). Bee pollen, except for 0.1 g/L dose in Palomino fino, moves forward the time to reach the maximum viable yeast populations (Figure 3). For Palomino fino variety, time differences are less pronounced, reaching between days 6 and 9 during AF with bee pollen addition compared to control (9th day). In Riesling variety, those maximum yeasts populations were reached between days 6 and 7, while control reached it about the 9th day. Generally, in all cases, bee pollen addition entails an increase in the maximum *Saccharomyces cerevisiae* population achieved.

**Figure 4:**
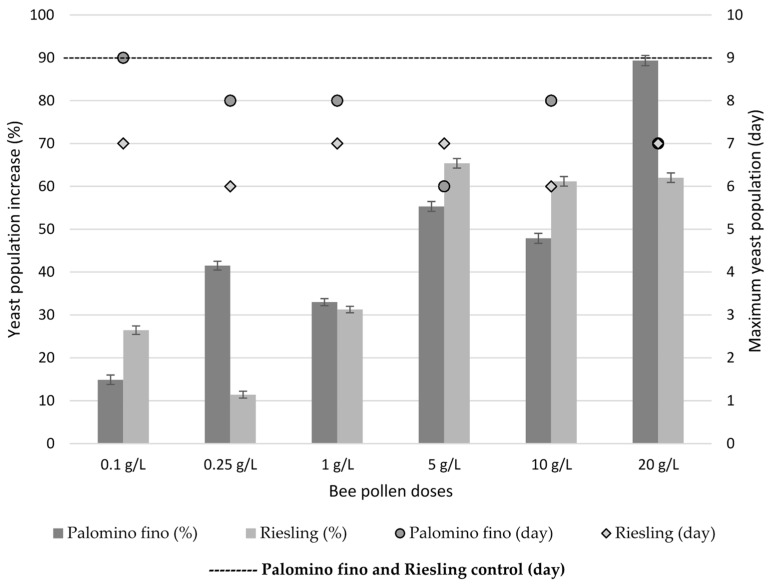
Yeast populations increase during alcoholic fermentation with bee pollen addition (0.1, 0.25, 1, 5, 10 and 20 g/L) compared to control.

Regarding to bee pollen dose, a greater increase in yeast populations with doses between 5 g/L and 20 g/L (from 47.9% to 89.4% for Palomino fino and from 61.2% to 65.4% for Riesling) was observed compared with low doses between 0.1 g/L and 1 g/L (from 14.9% to 41.5% for Palomino fino and from 11.4% to 31.3% for Riesling) (Figure 4).

### 2.4. YAN Evolution during AF

**Figure 5:**
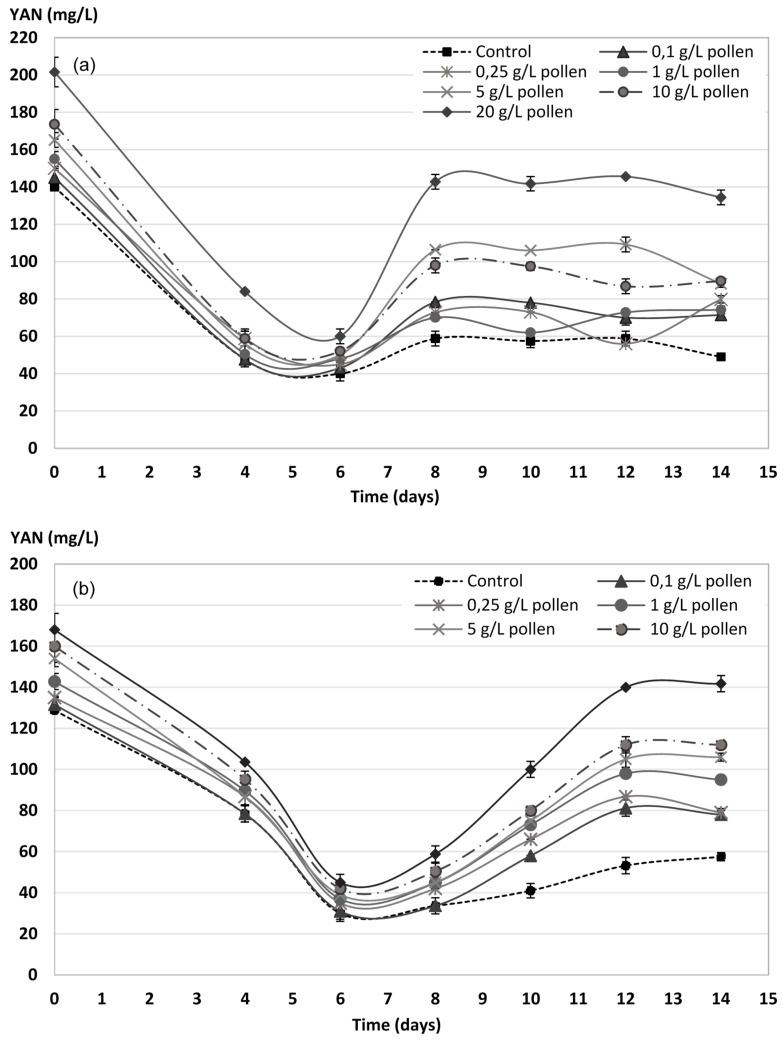
Evolution of the yeast-assimilable nitrogen (YAN) by *Saccharomyces cerevisiae* yeasts during the AF process of Palomino fino (**a**) and Riesling (**b**) grape musts with bee pollen doses. The results are the mean ± SD of three repetitions.

**Figure 6:**
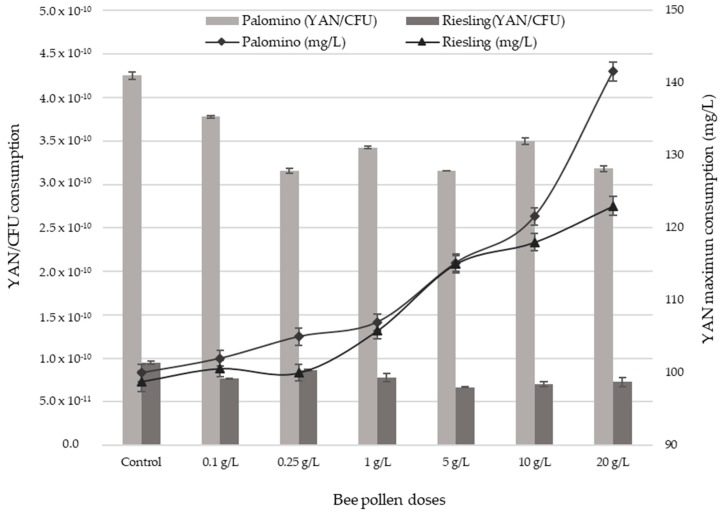
Maximum consumption of YAN against the maximum consumption of YAN/CFU during exponential growth phase of Palomino fino and Riesling grape musts with different bee pollen doses and control.

Figure 5a,b show YAN evolution in Palomino and Riesling musts, respectively, during AF. Both musts show YAN decrease in the first 6 days, coinciding with exponential growth phase end (Figure 3). YAN consumption increases with pollen dose (Figure 5) due to the maximum yeasts populations increase reached in each case (Figure 3). However, although YAN consumption showed some correlation with the maximum populations reached (r^2^ = 0.84 and 0.87 for Palomino and Riesling respectively), YAN consumption per colony forming unit CFU decreases significantly by an average of 20% with the addition of pollen compared to the control (Figure 6).

However, from the sixth day onwards there is an increase of the YAN until the eighth day for all the Palomino fino musts and until the twelfth day for the Riesling musts, coinciding with the death phase (Figure 3a,b). Control musts experience a slight increase in both cases. In addition, except for some doses in Palomino, it can be observed that YAN increases in greater proportion as the bee pollen dose applied increase, and is much more significant and prolonged in Riesling musts.

### 2.5. Physico-Chemical and Colour Parameters in Final Wines

Table 3 and Table 4 show the bee pollen influence on the physico-chemical parameters of wines obtained with the Palomino fino and Riesling varieties. Firstly, it can be observed there was non-significant effect on ethanol results. Nevertheless, volatile acidity, presented a significant increase in their values from the bee pollen dose of 10 g/L respect to control, while at small doses (between 0.1 and 5 g/L) no effect was observed. These increases could be caused mainly by little contributions of sugars at high bee pollen doses (Table 1 and Table 2). However, from the oenological point of view, these increases do not sensorially affect the quality of the wines.

In relation to the total acidity and pH, no clear significant influence with the doses of pollen was observed. Only at high doses (20 g/L) is there a slight decrease and increase in the total acidity and pH, respectively. This behaviour could be attributed to potassium (K) contribution by pollen, which is only appreciable at high doses in a proportion of 4 g/kg [29].

Mean values and standard deviation of the CIELab coordinates, a* (red/ green), b* (yellow/blue) and L* (lightness), colour saturation C* (chroma) and H* (hue), for the samples classified by of bee pollen doses (control, from 0.1 to 20 g/L), are shown in Table 3 and Table 4. As the tables show, there was a significant increase in the values of coordinates a* (red/green), b* (yellow/blue) and colour saturation C* (Chroma), from 1 g/L bee pollen dose in both wines Palomino fino and Riesling, compared to the control. These alterations caused significant displacement of wines in the CIELab coordinates corresponding to intense and dark orange tones. While, control and low pollen doses of bee pollen wines kept located within yellow tones, typical in white young wines. Also, the results showed a significant increase in the colour intensity and the absorbance at 420 nm (yellow), from 1 g/L of bee pollen. This can negatively affect the visual quality of white wine, giving a much rustier appearance in final wines. Finally, there was no significant variations in L* (luminosity) and H* (hue) observed in final wines.

## 3. Discussion

According to bee pollen composition data provided by the literature [36], a dose of 20 g/L could contribute to increase, glucose and fructose, in must between 5 and 10 g/L. Total acidity values at the different doses of pollen fluctuate, respect to control values (7.9 and 4.8 g/L for Palomino and Riesling respectively). However, honey-wines [30] noted an increase in total acidity by 0.02 g in tartaric acid per gram of pollen in honey must. This effect is not observed in grape must, possibly because its total acidity is on the order of 80% higher than in the honey must. From an oenological point of view, this is important since in wines there is a need to increase the total SO2 dose to prevent the enzymatic oxidations of the polyphenols [37] and the development of bacteria and yeasts that are sensitive to this antiseptic [38]. More acidic pH of Palomino fino grape must (3.11), compared with Riesling (3.34), could favor YAN dissolution from the solid matrix and enrich the grape must in greater measure. YAN represents nitrogen that can be easily assimilated by yeasts for cell growth [16,18,39,40] and as a survival factor in fermentation [2,40]. Therefore, pollen is a good source of YAN and can be used as a natural additive to enrich deficient musts, thus improving its fermentative potential. This effect was also verified for mead [30], although the doses of pollen used were higher (up to 50 g/L) due to the YAN deficiencies of honey musts. Several authors indicate bee pollen YAN is mostly composed of amino acids, representing 1/10 of the total nitrogen [29,41]. Some research works of pollen composition show that free amino acids concentration is between 14–16% (w/w), especially highlighting proline, glutamic acid, aspartic acid, lysine and leucine [26,30]. These amino acids could be of interest as growth-activating substances for yeast in alcoholic fermentation.

Differences in initial sugar concentration may be responsible in Riesling musts, regardless of pollen dose and YAN, for the increase of yeast populations to a greater extent during the exponential growth phase. This could increase sugars consumption in both, time and alcoholic fermentation speed. YAN increase, even at the highest doses, does not produce a reduction in the latency time and an improvement in the adaptation of the yeasts in the case of Riesling. However, in Palomino fino, which has a lower initial sugar content, pollen addition makes the populations slightly larger during lag phase compared to the control. Therefore, in this case, YAN content increase contributed by high pollen doses can, in turn, increase yeast inoculum survival during the latency phase. However, during the exponential growth phase, multiplication rate and maximum populations of viable yeasts are significantly higher in the Riesling musts (1.1 × 10^9^–1.67 × 10^9^ CFU/mL) than in the Palomino musts (2.4 × 10^8^–4.5 × 10^8^ CFU/mL) (Figure 3a,b), due to its higher sugar content. Therefore, pollen could contribute with other growth-activating substances than YAN to the yeast populations, but there was not reflected in fermentation kinetics at low bee pollen doses behaviour (Figure 1 and Figure 2).

Likewise, during stationary and death phases, yeast populations in the Palomino and Riesling musts remain much higher for all the pollen doses (ANOVA *p* < 0.05) than in control, especially at high doses. Bee pollen have an important effect on the survival of yeasts population during death phase. YAN consumption is not proportional with the maximum populations reached during AF, especially in Riesling. This fact could indicate that pollen addition to grape must reduce yeast’s YAN requirements. This could be because the pollen, in addition to providing YAN, is also providing other growth-activating substances, such as fatty acids. Several authors indicate that pollen is rich in polyunsaturated fatty acids such as linoleic and linolenic acid [28], which can be metabolized by yeasts during fermentation, favouring their growth [42].

Ethanol produced during alcoholic fermentation probably favours YAN extraction from pollen. In addition, yeast populations increase could contribute to YAN increase due to yeasts autolysis during cell lysis phase [43,44,45,46]. This would justify that, Riesling musts with higher alcohol content (12% *v*/*v*), reached higher YAN values at the end of fermentation. Moreover, it has to be pointed out that these residual YAN values could be of interest in the development of malolactic fermentation or biological ageing under the “flor” yeast veil. On the other hand, high bee pollen doses could generate microbiological instability, such as acetic bacteria or *Brettanomyces*, and ethyl carbamate accumulation in wine [9].

Bee pollen contribution of polyphenolic and pigmented compounds, such as flavonoids, leukotrienes, catechins, phenolic acids (1.6%) and β-carotene (0.07%) [47] could be responsables of colour parameter modifications in white young wines. Same behaviour was observed in the elaboration of mead with pollen, where absorbance measurements at 420 nm (yellow), increased with bee pollen increasing [30].

## 4. Materials and Methods

### 4.1. Winemaking Conditions

Palomino fino grape must was obtained from an Andalusian cooperative-winery, “Unión de Viticultores Chiclaneros” of “Chiclana de la Frontera” (Cádiz, Spain), and Riesling grape must provide by a winery in Arcos de la Frontera (Cádiz, Spain). Grape musts were transported in 25 L plastic food carafes from the winery cellar where the grapes were destemmed, ground and pressed. The must was obtained without adding sulfurous anhydride and stored at −20 °C until the beginning of the experiments. After the grape must was thawed and tempered at approximately 20 °C, potassium metabisulfite (up to 80–90 mg/L) (Sigma-Aldrich Chemical S.A., Madrid, Spain) was added, and the pH was corrected to a value of 3.2–3.3 by adding tartaric acid (Sigma-Aldrich Chemical S.A.). The sulphited grape must was then racked by gravity in methacrylate reservoirs at a temperature of 10 °C for 24 h. Once cleaned, grape must was homogenized and distributed in glass fermenters (V = 5 L) for each bee pollen doses and control with cooling jackets to control the temperature during laboratory-scale alcoholic fermentation assays. All the materials in contact with the samples during AF were decontaminated previously using peracetic acid and hydrogen peroxide mix.

### 4.2. Experimental Layout

Commercial bee pollen (Valencia, Spain) with 6% humidity and 99.8% dry-weight purity was added to must using six different doses: 0 (control), 0.1 and 0.25 g/L (low doses), 1 and 5 g/L (intermediate doses), and 10 and 20 g/L (high doses) and homogenized. Before use, the pollen was ground in a mill (Vorwerk’s Thermomix TM31, Wuppertal, Germany), then stored in a dark glass bottle under desiccator (low humidity and vacuum) conditions. All trials were performed in triplicate (n = 3) to ensure statistical significance. Palomino fino and Riesling grape musts were inoculated with a commercial active dry wine yeast (ADWY) strain of *Saccharomyces cerevisiae* Lalvin 71B^®^ (Lallemand, Barcelona, Spain), at a dose of 10 g/hL and incubated at 19–20 °C (6.7 × 10^6^ CFU/mL). Once the alcoholic fermentation was complete (stable density measurement and the residual reducing sugars below 2 g/L), wines were chilled (6 °C) for 1–2 days and subsequently treated with gelatin (4 g/hL) and bentonite (40 g/hL). Finally, all wines were filtered (1.2 µm and 0.22 µm membrane filters) (HAWP Millipore Co., Bedford, MA, USA) and bottled using nitrogen pressure.

### 4.3. Analytical Measurements

Physico-chemical parameters of all vinifications (with bee pollen addition (from 0.1 to 20 g/L) and control) including the °Bè (initial grape musts), alcoholic content (%), pH, total acidity (TA), volatile acidity (VA), yeast-assimilable nitrogen content (YAN), and the sugars (RS), glucose and fructose, were analyzed. In addition, chromatic characteristics of wines were evaluated by analyzing the parameters CIELab (L*, a*, b*, H* and C*), colour intensity and absorbance at 420 nm.

°Bè was determined using a calibrated Dujardin-Salleron hydrometer (Laboratories Dujardin-Salleron, Arcueil Cedex, France), alcohol content, total acidity and volatile acidity were determined according to the official methods of wine analysis [48]. pH measurement was carried out using a digital pH-meter CRISON-2001^®^ (Crison, Barcelona, Spain) equipped with a combined electrode with automatic temperature compensation. Yeast-assimilable nitrogen (YAN) was determined according to the formol method described by Aerny [49].

Residual sugars (RS) (glucose and fructose) in final wines were analyzed by ion chromatography (930 Compact IC Flex, Metrohm, Herisau, Switzerland) with a pulse amperometric detector and a gold electrode as the working electrode. Elution was carried out isocratically at a 0.5 mL/min flow rate with 300 mM sodium hydroxide (NaOH) and 1 mM sodium acetate (NaOAc). Separation was achieved on a Metrosep Carb 2–150/4.0 column (Metrohm).

The entire visible spectrum (380–780 nm) was recorded (Δλ = 5 nm) using an Illuminant D65 (daylight source) in 10 mm glass cuvettes with a HITACHI UV-Visible 2001 spectrophotometer (PACISA Y GIRALT, SL, Grupo TAPER, Madrid, Spain) and a 10° observer (perception angle of the human observer) as standard conditions. Colour evaluation was carried out following the recommendations of the International Commission of L’Eclairage, which consider that the CIELAB parameters better define the colour of the wine and allow a better differentiation [50,51,52]. Colour intensity was determined by the rapid method recommended by the Office International de la Vigne et du Vin, which is the same as that proposed by Sudraud based on the sum of the absorbance at 420 and 520 nm (Recueil des méthodes internationales d’analyze Des vins et des moûts, 41-58, 1990). Absorbance measurements at 420 nm were established using a UV-Visible HITACHI spectrophotometer model U-2001 (PACISA Y GIRALT, S.L). Before measurements, all samples were filtered using 0.45 µm nylon syringe membrane filters (HAWP Millipore Co.).

### 4.4. Yeast (Saccharomyces cerevisiae)

Yeast population counts were performed using a Neubauer chamber (Brand^®^, Merck, Madrid, Spain) with methylene blue staining method to differentiate between the viable and non-viable cells. For the counts, a microscope with 40× magnification (Nikon, Tokyo, Japan) was used.

### 4.5. Statistical Analysis

Means and standard deviations were calculated, and significant differences were evaluated by two-way ANOVA and Bonferroni’s multiple range (BSD) test; *p* < 0.05 was considered significant (GraphPad Prism version 6.01 for Windows, GraphPad Software, San Diego, CA, USA).

## 5. Conclusions

Bee pollen addition (0.1, 0.25, 1, 5, 10 and 20 g/L doses) produces significant increases in YAN and maximum yeasts population reached during AF in Palomino fino and Riesling grape musts. From the kinetic point of view, V_exp_ increase significantly from 1 g/L and 5 g/L, in Palomino and Riesling respectively. Bee pollen have an important effect on yeasts survival during death phase. Final wines showed significantly increase in volatile acidity from 10 g/L and, CIELab parameters (a*, b* y C*), colour intensity and Abs 420 nm, from 1 g/L. Therefore, pollen could be used as a natural alternative as fermentative activator for the alcoholic fermentation of white wines, using dosages less than 1 g/L.

## Figures and Tables

**Table 1:** Physico-chemical parameters of Palomino fino grape must with addition of bee pollen (0.1, 0.25, 1, 5, 10 and 20 g/L) and control.

Parameters	Palomino Fino Grape Must Bee Pollen Doses																				
	Control			0.1 g/L			0.25 g/L			1 g/L			5 g/L			10 g/L			20 g/L		
°Bè	10.03	±	0.10 ^a,b^	9.70	±	0.02 ^a^	9.95	±	0.04 ^a^	10.00	±	0.03 ^a^	10.05	±	0.05 ^a,b^	10.10	±	0.05 ^a,b^	10.55	±	0.03 ^b^
Density (g/cm^3^) at 20 °C	1.070	±	0.000 ^a^	1.070	±	0.000 ^a^	1.070	±	0.000 ^a^	1.070	±	0.000 ^a^	1.071	±	0.000 ^a^	1.071	±	0.000 ^a^	1.073	±	0.000 ^a^
Sugars (g/L)	165.17	±	0.64 ^a^	159.30	±	0.37 ^a^	163.70	±	0.34 ^a^	164.80	±	0.25 ^a^	164.80	±	0.21 ^a^	168.10	±	0.18 ^a,b^	175.67	±	0.58 ^b^
Total acidity (g/L TH_2_)	7.09	±	0.07 ^a,b,c^	6.87	±	0.12 ^a^	6.97	±	0.03 ^a,b^	7.22	±	0.07 ^a,b,c^	7.24	±	0.05 ^a,b,c^	7.26	±	0.05 ^b,c^	7.46	±	0.10 ^c^
pH	3.11	±	0.01 ^a^	3.10	±	0.01 ^a^	3.10	±	0.01 ^a^	3.11	±	0.01 ^a^	3.11	±	0.01 ^a^	3.11	±	0.01 ^a^	3.18	±	0.01 ^a^
Free SO_2_	24	±	1 ^a^	23	±	1 ^a^	24	±	1 ^a^	24	±	1 ^a^	24	±	1 ^a^	24	±	2 ^a^	27	±	1 ^b^
Total SO_2_	70	±	2 ^a^	68	±	1 ^a^	70	±	2 ^a^	70	±	2 ^a^	70	±	1 ^a^	70	±	3 ^a^	74	±	1 ^b^
YAN (mg/L)	140	±	1 ^a^	144	±	3 ^a,b,c^	151	±	0 ^b,c^	154	±	2 ^c^	165	±	4 ^d^	184	±	8 ^e^	202	±	8 ^f^

Different lowercase superscript letters mean statistically significant differences between samples at *p* < 0.05 obtained by two-way ANOVA and Bonferroni’s multiple range (BSD) test. Total acidity expressed in tartaric acid (TH_2_). Results are the means ± SD of three repetitions.

**Table 2:** Physico-chemical parameters of Riesling grape must with addition of bee pollen (0.1, 0.25, 1, 5, 10 and 20 g/L) and control.

Parameters	Riesling Grape Must Bee Pollen Doses																				
	Control			0.1 g/L			0.25 g/L			1 g/L			5 g/L			10 g/L			20 g/L		
°Bè	11.07	±	0.06 ^a^	10.87	±	0.06 ^a^	11.02	±	0.06 ^a^	11.27	±	0.06 ^a^	11.27	±	0.01 ^a^	11.37	±	0.06 ^a^	11.62	±	0.06 ^a^
Density (g/cm^3^) at 20 °C	1.081	±	0.000 ^a^	1.081	±	0.000 ^a^	1.080	±	0.000 ^a^	1.082	±	0.000 ^a^	1.082	±	0.000 ^a^	1.083	±	0.000 ^a^	1.084	±	0.000 ^a^
Sugars (g/L)	189.00	±	0.00 ^a^	189.70	±	0.00 ^a^	187.40	±	0.00 ^a^	191.80	±	0.00 ^a,b^	191.80	±	0.00 ^a,b^	195.13	±	0.06 ^a,b^	204.83	±	0.06 ^b^
Total acidity (g/L TH_2_)	4.83	±	0.24 ^a^	4.80	±	0.26 ^a^	4.91	±	0.21 ^a^	4.89	±	0.24 ^a^	4.99	±	0.23 ^a^	4.81	±	0.22 ^a^	4.76	±	0.21 ^a^
pH	3.34	±	0.01 ^a^	3.34	±	0.01 ^a^	3.34	±	0.01 ^a^	3.34	±	0.00 ^a^	3.35	±	0.00 ^a^	3.33	±	0.01 ^a^	3.33	±	0.01 ^a^
Free SO_2_	17	±	0 ^a^	17	±	1 ^a^	17	±	1 ^a^	18	±	1 ^a^	17	±	1 ^a^	17	±	1 ^a^	17	±	1 ^a^
Total SO_2_	60	±	2 ^a^	62	±	1 ^a^	61	±	1 ^a^	60	±	1 ^a^	61	±	1 ^a^	60	±	2 ^a^	61	±	1 ^a^
YAN (mg/L)	128	±	0 ^a^	132	±	1 ^a,b^	140	±	1 ^b,c^	146	±	4 ^c,d^	154	±	2 ^d,e^	160	±	3 ^e,f^	168	±	3 ^f^

Different lowercase superscript letters mean statistically significant differences between samples at *p* < 0.05 obtained by two-way ANOVA and Bonferroni’s multiple range (BSD) test. Total acidity expressed in tartaric acid (TH_2_). Results are the means ± SD of three repetitions.

**Table 3:** Physico-chemical parameters of Palomino fino wines with bee pollen addition (0.1, 0.25, 1, 5, 10 and 20 g/L) and control.

Parameters	Palomino Fino Wines Bee Pollen Doses																				
	Control			0.1 g/L			0.25 g/L			1 g/L			5 g/L			10 g/L			20 g/L		
% Alcohol *v*/*v*	9.81	±	0.03 ^a^	9.78	±	0.01 ^a^	9.72	±	0.01 ^a^	9.73	±	0.02 ^a^	9.52	±	0.02 ^a^	9.91	±	0.02 ^a^	10.08	±	0.02 ^a^
pH	2.99	±	0.01 ^a^	3.01	±	0.02 ^a^	3.01	±	0.01 ^a^	3.01	±	0.01 ^a^	3.04	±	0.01 ^a^	3.04	±	0.01 ^a^	3.13	±	0.01 ^a^
Total acidity (g/L)	6.24	±	0.16 ^a^	6.01	±	0.03 ^a^	6.08	±	0.01 ^a^	6.27	±	0.03 ^a^	6.44	±	0.02 ^a^	6.21	±	0.02 ^a^	5.97	±	0.02 ^a^
Volatile acidity (g/L)	0.10	±	0.01 ^a^	0.11	±	0.01 ^a^	0.10	±	0.01 ^a^	0.10	±	0.01 ^a^	0.10	±	0.01 ^a^	0.15	±	0.01 ^b^	0.24	±	0.01 ^c^
Glucose (g/L)	0.214	±	0.016 ^a^	0.187	±	0.017 ^b^	0.190	±	0.020 ^b^	0.170	±	0.020 ^c^	0.110	±	0.001 ^d^	0.110	±	0.001 ^d^	0.130	±	0.002 ^e^
Fructose (g/L)	0.468	±	0.019 ^a^	0.194	±	0.007 ^b^	0.190	±	0.010 ^b^	0.380	±	0.040 ^c^	0.420	±	0.002 ^d^	0.370	±	0.001 ^c^	0.150	±	0.010 ^e^
L*	98.97	±	0.04 ^a^	98.85	±	0.16 ^a^	98.79	±	0.07 ^a^	98.63	±	0.10 ^a^	98.51	±	0.08 ^a^	98.47	±	0.06 ^a^	97.84	±	0.05 ^a^
a*	1.02	±	0.03 ^a^	1.06	±	0.03 ^a^	1.07	±	0.02 ^a,b^	1.16	±	0.03 ^b^	1.28	±	0.01 ^c,d^	1.35	±	0.02 ^d^	1.65	±	0.02 ^e^
b*	5.92	±	0.04 ^a^	6.05	±	0.08 ^a^	6.17	±	0.03 ^a^	6.86	±	0.05 ^b^	7.50	±	0.07 ^c,d^	7.83	±	0.01 ^d^	10.09	±	0.01 ^e^
H*	99.71	±	0.32 ^a^	99.92	±	0.40 ^a^	99.57	±	0.15 ^a^	99.55	±	0.25 ^a^	99.64	±	0.04 ^a^	99.81	±	0.12 ^a^	99.28	±	0.08 ^a^
C*	6.01	±	0.04 ^a^	6.14	±	0.07 ^a^	6.47	±	0.03 ^a,b^	6.96	±	0.05 ^b^	7.61	±	0.07 ^c,d^	7.95	±	0.01 ^d^	10.22	±	0.02 ^e^
Color Intensity	0.11	±	0.00 ^a^	0.11	±	0.01 ^a^	0.12	±	0.01 ^a^	0.14	±	0.00 ^b^	0.16	±	0.01 ^c^	0.18	±	0.01 ^d^	0.21	±	0.01 ^e^
Abs. 420	0.09	±	0.00 ^a^	0.09	±	0.01 ^a^	0.10	±	0.01 ^a,b^	0.11	±	0.00 ^b,c^	0.12	±	0.00 ^c,d^	0.13	±	0.00 ^d^	0.17	±	0.01 ^e^

Different lowercase superscript letters mean statistically significant differences between samples at *p* < 0.05 obtained by two-way ANOVA and Bonferroni’s multiple range (BSD) test. Results are the means ± SD of three repetitions.

**Table 4:** Physico-chemical parameters of Riesling wines with bee pollen addition (0.1, 0.25, 1, 5, 10 and 20 g/L) and control.

Parameters	Riesling Grape Wines Bee Pollen Doses																				
	Control			0.1 g/L			0.25 g/L			1 g/L			5 g/L			10 g/L			20 g/L		
% Alcohol *v*/*v*	12.47	±	0.15 ^a^	12.67	±	0.05 ^a^	12.67	±	0.05 ^a^	12.63	±	0.05 ^a^	12.57	±	0.05 ^a^	12.70	±	0.10 ^a^	12.77	±	0.05 ^a^
pH	3.39	±	0.01 ^a^	3.39	±	0.01 ^a^	3.39	±	0.01 ^a^	3.38	±	0.01 ^a^	3.43	±	0.01 ^a^	3.47	±	0.05 ^a^	3.57	±	0.02 ^a^
Total acidity (g/L)	3.98	±	0.03 ^a^	3.94	±	0.07 ^a^	4.06	±	0.03 ^a^	4.04	±	0.08 ^a^	4.09	±	0.08 ^a^	3.96	±	0.01 ^a^	3.90	±	0.01 ^a^
Volatile acidity (g/L)	0.25	±	0.01 ^a^	0.23	±	0.01 ^b^	0.23	±	0.01 ^b^	0.18	±	0.00 ^c^	0.13	±	0.01 ^d^	0.28	±	0.01 ^e^	0.32	±	0.01 ^f^
Glucose (g/L)	0.028	±	0.001 ^a^	0.194	±	0.003 ^b^	0.207	±	0.003 ^c^	0.228	±	0.002 ^d^	0.227	±	0.000 ^d^	0.246	±	0.002 ^d^	0.251	±	0.002 ^d^
Fructose (g/L)	0.083	±	0.004 ^a^	0.436	±	0.005 ^b,c^	0.432	±	0.003 ^c^	0.395	±	0.002 ^d^	0.264	±	0.002 ^e^	0.259	±	0.000 ^e^	0.252	±	0.024 ^e^
L*	98.98	±	0.02 ^a^	99.16	±	0.01 ^a^	99.19	±	0.03 ^a^	99.16	±	0.03 ^a^	98.69	±	0.07 ^a^	97.49	±	0.01 ^a^	97.52	±	0.04 ^a^
a*	0.59	±	0.02 ^a^	0.53	±	0.01 ^a^	0.54	±	0.02 ^a^	0.75	±	0.02 ^b^	0.91	±	0.01 ^c^	1.26	±	0.01 ^d^	2.03	±	0.01 ^e^
b*	4.10	±	0.02 ^a^	4.20	±	0.01 ^a^	4.30	±	0.01 ^a^	4.95	±	0.02 ^b^	6.54	±	0.05 ^c^	8.58	±	0.02 ^d^	12.74	±	0.02 ^e^
H*	97.44	±	0.29 ^a^	97.25	±	0.25 ^a^	97.7	±	0.36 ^a^	97.81	±	0.26 ^a^	97.9	±	0.16 ^a^	98.4	±	0.08 ^a^	99.1	±	0.08 ^a^
C*	4.53	±	0.02 ^a^	4.24	±	0.01 ^a^	4.39	±	0.01 ^a^	4.80	±	0.03 ^a^	6.61	±	0.05 ^b^	8.58	±	0.02 ^c^	12.9	±	0.02 ^d^
Color Intensity	0.08	±	0.01 ^a^	0.07	±	0.01 ^a^	0.07	±	0.01 ^a^	0.10	±	0.01 ^b^	0.12	±	0.01 ^c^	0.19	±	0.01 ^d^	0.25	±	0.01 ^e^
Abs. 420	0.06	±	0.01 ^a^	0.06	±	0.01 ^b,c^	0.06	±	0.01 ^c^	0.10	±	0.01 ^d,e^	0.10	±	0.01 ^e^	0.14	±	0.01 ^f^	0.21	±	0.01 ^g^

Different lowercase superscript letters mean statistically significant differences between samples at *p* < 0.05 obtained by two-way ANOVA and Bonferroni’s multiple range (BSD) test. Results are the means ± SD of three repetitions.
